# Lysyl oxidase like 2 is increased in asthma and contributes to asthmatic airway remodelling

**DOI:** 10.1183/13993003.04361-2020

**Published:** 2022-07-07

**Authors:** Jopeth Ramis, Robert Middlewick, Francesco Pappalardo, Jennifer T. Cairns, Iain D. Stewart, Alison E. John, Shams-Un-Nisa Naveed, Ramaswamy Krishnan, Suzanne Miller, Dominick E. Shaw, Christopher E. Brightling, Lee Buttery, Felicity Rose, Gisli Jenkins, Simon R. Johnson, Amanda L. Tatler

**Affiliations:** 1 Biodiscovery Institute, University of Nottingham, Nottingham, UK; 2 Dept of Chemical Engineering, Technological Institute of the Philippines, Manila, Philippines; 3 Centre for Respiratory Research/NIHR Biomedical Research Centre, School of Medicine, University of Nottingham, Nottingham, UK; 4 Margaret Turner Warwick Centre for Fibrosing Lung Disease, National Heart and Lung Institute, Imperial College London, London, UK; 5 Institute for Lung Health, Leicester NIHR Biomedical Research Centre, University of Leicester, Leicester, UK; 6 Center for Vascular Biology Research, Beth Israel Deaconess Medical Center, Harvard Medical School, Boston, MA, USA

## Abstract

**Background:**

Airway smooth muscle (ASM) cells are fundamental to asthma pathogenesis, influencing bronchoconstriction, airway hyperresponsiveness and airway remodelling. The extracellular matrix (ECM) can influence tissue remodelling pathways; however, to date no study has investigated the effect of ASM ECM stiffness and cross-linking on the development of asthmatic airway remodelling. We hypothesised that transforming growth factor-β (TGF-β) activation by ASM cells is influenced by ECM in asthma and sought to investigate the mechanisms involved.

**Methods:**

This study combines *in vitro* and *in vivo* approaches: human ASM cells were used *in vitro* to investigate basal TGF-β activation and expression of ECM cross-linking enzymes. Human bronchial biopsies from asthmatic and nonasthmatic donors were used to confirm lysyl oxidase like 2 (LOXL2) expression in ASM. A chronic ovalbumin (OVA) model of asthma was used to study the effect of LOXL2 inhibition on airway remodelling.

**Results:**

We found that asthmatic ASM cells activated more TGF-β basally than nonasthmatic controls and that diseased cell-derived ECM influences levels of TGF-β activated. Our data demonstrate that the ECM cross-linking enzyme LOXL2 is increased in asthmatic ASM cells and in bronchial biopsies. Crucially, we show that LOXL2 inhibition reduces ECM stiffness and TGF-β activation *in vitro*, and can reduce subepithelial collagen deposition and ASM thickness, two features of airway remodelling, in an OVA mouse model of asthma.

**Conclusion:**

These data are the first to highlight a role for LOXL2 in the development of asthmatic airway remodelling and suggest that LOXL2 inhibition warrants further investigation as a potential therapy to reduce remodelling of the airways in severe asthma.

## Abstract 2

**Novel role for matrix cross-linking enzyme LOXL2 in asthmatic airway remodelling: LOXL2 is increased in #asthma but LOXL2 inhibition reduces matrix stiffness in airway smooth muscle cells and reduces remodelling *in vivo*** https://bit.ly/3FnzGb3

## Introduction

Airway remodelling is a common feature in asthma, and can include epithelial shedding, increased airway smooth muscle (ASM) mass and subepithelial fibrosis. The extracellular matrix (ECM) is emerging as a key modulator of inflammatory and remodelling events [1–3]. Proteins present within asthmatic ECM can influence the secretory profile of ASM cells and promote their proliferation [4, 5], and influence airway remodelling, hyperresponsiveness and inflammation [6]. The relative stiffness of the ECM can also influence cell behaviour [7, 8]. In the lung, increased ECM stiffness initiates epithelial–mesenchymal transition and causes fibroblasts to synthesise collagen [9, 10]. Furthermore, increased matrix stiffness can increase ASM cell proliferation and contractile force [11, 12]. Taken together, these studies suggest that increased ECM stiffness may contribute to the development of airway remodelling.

ECM stiffness is influenced by cross-linking, the formation of covalent bonds between ECM proteins, which is catalysed by ECM cross-linking enzymes. Lysyl oxidase like 2 (LOXL2) belongs to the lysyl oxidase family of enzymes, and is responsible for cross-linking and stabilising collagen fibres. Increased LOXL2 expression is evident in various fibrotic diseases [13–17] and LOXL2 inhibition reduces fibrosis in multiple animal models of disease [17–20]. To date, there have been no studies investigating a role for LOXL2 in asthma pathogenesis or airway remodelling.

This study investigated a role for ECM alterations and LOXL2 in the development of asthmatic airway remodelling. We found that activation of transforming growth factor-β (TGF-β) by ASM cells is influenced by the ECM, and that ASM cells isolated from asthmatic donors exhibit enhanced basal TGF-β activation and deposit a stiffer ECM. We show that LOXL2 expression is increased in asthma, and that LOXL2 inhibition reduces ASM cell ECM stiffness and TGF-β activation *in vitro* and reduces airway remodelling in an *in vivo* asthma model.

## Materials and methods

### Cell culture

The source of human ASM cells is detailed in the supplementary material. ASM cells were cultured as previously described [21] and growth arrested in serum-free DMEM for 24 h prior to experiments. A minimum of three donor cell lines were used in each experiment.

### Culture of ASM cells on methacrylated gelatin hydrogels

A hydrogel disc was produced from a precursor gelatin methacrylol (GelMA) solution containing 0.5% Irgacure 2959 by cross-linking under UV light (365 nm). Solutions of 5, 10 and 15% w/v GelMA were dissolved to obtain hydrogels that were 1×, 6× and 12× that of the physiological stiffness of tracheal smooth muscle reported previously [22]. Hydrogel relative stiffness (*E*) was determined by a TA.HDplus texture analyser (Stable Micro Systems, Godalming, UK) (supplementary figure S1). ASM cells were seeded at 2×10^4^ cells·cm^−2^ and cultured for 6 days.

### Phosphorylated Smad2 immunofluorescence

Cells were fixed (4% paraformaldehyde), permeabilised (0.3% Triton-X-100), and then stained with phosphorylated Smad2 (p-Smad2) primary antibody (1:100) and an Alexa Fluor 488 antibody (1:3000). Nuclei were stained with 4′,6-diamidino-2-phenylindole. Image analysis was performed using a Zeiss LSM 710 confocal microscope (Zeiss, Oberkochen, Germany) and Cell Profiler [23].

### Cyclical stretching

Fully confluent ASM cells on collagen I-coated BioFlex plates were stretched (15%, 0.3 Hz) using the Flexcell FX-6000T Tension System (Flexcell, Burlington, NC, USA). Additional plates were incubated alongside the Flexcell device to serve as unstretched controls.

### Western blotting

p-Smad2 Western blotting was performed as previously described [24]. To measure LOXL2 and LOXL3 expression, cell lysates were electrophoresed, transferred onto PVDF membrane, and then probed using antibodies against LOXL2 (1:1000), LOXL3 (1:2000) and GAPDH (1:3000). Bands were visualised using Clarity ECL (Bio-Rad, Hercules, CA, USA) and densitometry performed using ImageJ (https://imagej.nih.gov).

### Transformed mink lung epithelial cell assay

The transformed mink lung epithelial cell (TMLC) (a kind gift of Daniel Rifkin, NYU Grossman School of Medicine, New York, NY, USA) is a widely used TGF-β reporter cell that expresses luciferase in response to active TGF-β. TMLC co-culture assay was performed as previously described [24] using ASM cells seeded on 96-well plates at 6.289×10^4^ cells·cm^−2^.

### p-Smad2 ELISA

p-Smad2 in cell lysates was measured using an ELISA kit (Cell Signaling Technologies, Danvers, MA, USA) according to the manufacturer's instructions. Due to the absence of a standard for generation of a standard curve, data are presented as raw optical density values.

### Collagen gel contraction assay

Contractility was assessed using a collagen gel cell contraction assay (Cell Biolabs, San Diego, CA, USA) according to the manufacturer's instructions. Briefly, ASM cells were seeded at 0.5×10^6^ cells per well in 24-well plates (in line with the manufacturer's recommended cell density) and the gel diameter was quantified after 24 h using a Nikon SMZ1500 stereomicroscope (Nikon, Tokyo, Japan) and Image J. Positive and negative controls were performed using methacholine and 2,3-butanedione monoxime, respectively.

### Traction force microscopy

ASM cells were seeded (62 890 cells·cm^−2^) on 3 kPa polydimethylsiloxane NuSil GEL-8100-coated 96-well plates with embedded fluorescent microspheres [25]. Fluorescent bead displacement was used to calculate traction forces using the method of Fourier transform traction cytometry [26]. Traction force data were obtained from 8–24 separate wells per cell line. The root mean square value for traction (in Pa) was reported.

### ECM crossover experiments

ASM cells were seeded at 62 890 cells·cm^−2^, cultured for 3 days and then digested in 0.016 M ammonium hydroxide for 30 min. Nonasthmatic cells were seeded on either their own ECM or ECM deposited by asthmatic ASM cells at 62 890 cells·cm^−2^ in serum-free media and *vice versa* for asthmatic ASM cells. End-point analysis was performed 24 h later.

### Quantitative real-time reverse transcriptase-PCR

Gene expression changes were assessed using quantitative reverse transcriptase (qRT)-PCR as previously described [27]. Primer sequences are shown in supplementary table S2.

### Atomic force microscopy

A MFP-3D standalone atomic force microscope (Oxford Instruments, Abingdon, UK) was used to obtain force–displacement curves of the ECM for Young's modulus (*E*) calculation. Instrument sensitivity was calibrated with the unloading force–displacement curve slope on glass, while the instrument thermal fluctuations were used to extract the effective spring constant of the tip, inherent to its resonant frequency. For each measurement of the sample, at least 100 force–displacement curves were recorded.

### Bronchial biopsy collection

Bronchial biopsies obtained from asthmatic and healthy control donors (n=6 per group) were used to determine LOXL2 expression within ASM bundles. All tissue was collected at the Nottingham Biomedical Research Centre, University of Nottingham (Nottingham, UK), under informed, written consent with ethical approval (12/EM/0199). More details are provided in the supplementary material.

### Immunohistochemistry

Parallel 5 µm sections of lung tissue were boiled in citrate buffer and then incubated with either a LOXL2 (1:1000) or α-smooth muscle actin (α-SMA) (1:1000) antibody overnight followed by a goat anti-rabbit antibody (1:200). The sections were incubated with 3,3′-diaminobenzidine and counterstained with Mayer's haematoxylin. ImageJ was used to overlay positive LOXL2 staining on α-SMA regions to quantify percentage reactivity.

### LOXL2 inhibition in an *in vivo* ovalbumin model

Studies were approved by the University of Nottingham Animal and Welfare Ethical Review Board and performed under Home Office licence authority within the Animal (Scientific Procedures) Act 1986. Following sensitisation by intraperitoneal injection of ovalbumin (OVA)/alum, animals were randomised to one of four treatment groups: PBS+vehicle, PBS+PAT1251, OVA+vehicle or OVA+PAT1251. Full details of the model are provided in the supplementary material.

### Histology

Haematoxylin/eosin and Masson's trichrome staining of 5 µm thick lung sections was performed as previously described [28, 29]. Staining was visualised using a Nikon Eclipse 90i microscope and NIS Elements AR3.2 software (Nikon).

### Immunostaining

Murine lung tissue was subjected to α-SMA immunofluorescence and quantification of small airway (radius <100 µm) staining was performed as previously described [21].

### Statistical analysis

Data are reported as a median of n observations and nonparametric tests were used in all cases. All statistical tests were discussed with a statistician (I.D.S.). Further details are included in the supplementary material. Details of the statistical tests used are included in the figure legends.

## Results

### Substrate stiffness and cyclical stretch activates TGF-β in human ASM cells

**FIGURE 1:**
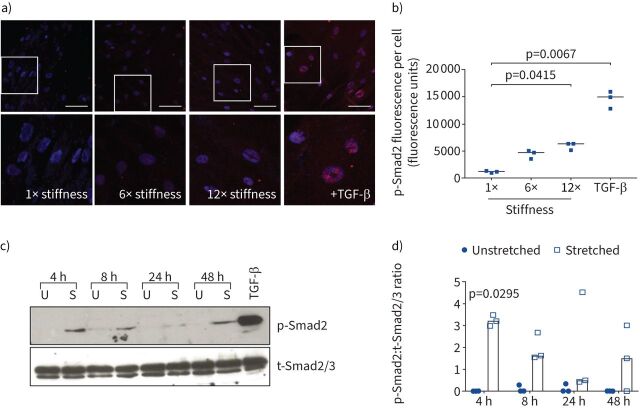
Matrix stiffness and cell stretching mediates transforming growth factor-β (TGF-β) activation. a) Representative images of airway smooth muscle (ASM) cells cultured on gelatin methacrylol substrates of increasing stiffness (1×, 6× and 12× that of physiological stiffness [22]) and stained for phosphorylated Smad2 (p-Smad2) and 4′,6-diamidino-2-phenylindole. TGF-β (5 ng·mL^−1^) was used as a positive control. Scale bar: 50 μm. Boxed area is shown at higher magnification to better visualise nuclear p-Smad2. b) Nuclear p-Smad2 in a) was quantified. Data are presented as median fluorescence units from cells of three individual donors. Cell number analysed was 106–305 per donor per condition. The experiment was independently repeated twice. The Kruskal–Wallis nonparametric test with Dunn's multiple comparison test was used. c) ASM cells were stretched (15%, 0.3 Hz (S)) or left unstretched (U). p-Smad2 and total Smad2/3 (t-Smad2/3) were measured by Western blotting. Blot shown is representative of n=3 donor cells. d) Densitometric analysis of the Western blots outlined in c) was performed and data are presented as median p-Smad2:t-Smad2/3 ratio. The Kruskal–Wallis nonparametric test with Dunn's multiple comparison test was used.

We have previously demonstrated that contractile agonists increase integrin-mediated TGF-β activation in human ASM cells [21]. Since both substrate stiffness and cyclical stretch can influence integrin-mediated TGF-β activation in epithelial cells [30, 31], we investigated whether these processes affect TGF-β activation in ASM cells. Human ASM cells were cultured on GelMA hydrogels of increasing stiffness for 7 days. The stiffness of the hydrogels was determined using a texture analyser (supplementary figure S1). Active TGF-β signalling (nuclear p-Smad2) was present when human ASM cells were cultured on a substrate of equivalent stiffness to the physiological stiffness of tracheal smooth muscle (denoted as 1×) [22] and levels increased with increased substrate stiffness (figure 1a and b). Additionally, cyclical mechanical stretch caused increased p-Smad2 levels after 4 h (figure 1c and d). Taken together, these data show that basal TGF-β activation by ASM cells can be influenced by both substrate stiffness and cyclical stretching.

### Asthmatic ASM cells activate increased levels of TGF-β

**FIGURE 2:**
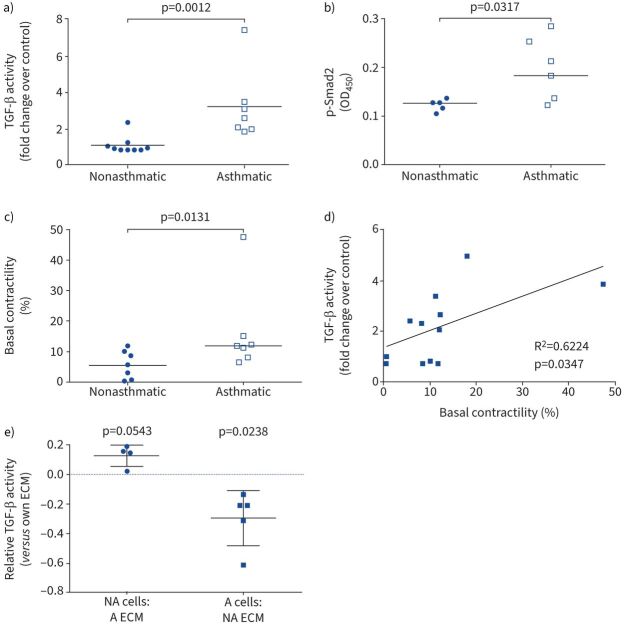
Basal transforming growth factor-β (TGF-β) activation is enhanced in asthma and influenced by the extracellular matrix (ECM). a) TGF-β activity in airway smooth muscle (ASM) cells was measured by the transformed mink lung epithelial cell (TMLC) reporter cell assay. Data are presented as median fold change in TGF-β activity in pg·mL^−1^ per 10^4^ cells *versus* the mean data for nonasthmatic cells. The nonparametric Mann–Whitney test was used. b) Basal phosphorylated Smad2 (p-Smad2) levels were measured by ELISA. Data are presented as median optical density at 450 nm (OD_450_). The nonparametric Mann–Whitney test was used. c) Cell contractility was measured using a collagen gel contraction assay. Data are presented as median percentage contraction of the collagen gel. The nonparametric Mann–Whitney test was used. d) Basal TGF-β activity in a) was correlated with basal cell contractility in c) using a Spearman correlation and the R^2^-value calculated. e) Nonasthmatic (NA) ASM cells were cultured on asthmatic (A) ECM and *vice versa* prior to determination of TGF-β activity by the TMLC assay. Data for cells from individual donors are presented as relative levels when cultured on the cell's own ECM. Blue dotted line denotes no change in TGF-β activity. Data are presented as mean±sd relative TGF-β activity. The one-sample t-test was used.

We next sought to investigate whether basal TGF-β activation by ASM cells is aberrant in asthma. Basal TGF-β activation was increased in ASM cells isolated from asthmatic donors compared with nonasthmatic cells as measured by a TMLC co-culture assay (figure 2a) and by p-Smad2 ELISA (figure 2b). To determine if increased TGF-β activation in asthmatic ASM cells was due to increased transcription of TGF-β genes we measured *TGFB1*, *TGFB2* and *TGFB3* mRNA levels. We found no significant difference in any of the three genes between asthmatic and nonasthmatic ASM cells (supplementary figure S1b–d). As we, and others, have shown a role for cell contractility in regulating TGF-β activation [21, 30, 32], we measured cell contractility. Contractility of asthmatic ASM cells was increased compared with nonasthmatic controls when measured using a collagen gel contraction assay (figure 2c) and the degree of contractility weakly correlated with the amount of TGF-β activated by the cells (figure 2d). However, cell contraction was similar between asthmatic and nonasthmatic ASM cells when measured by traction force microscopy (TFM) (supplementary figure S2a).

To further explore whether differences in cytoskeleton-mediated TGF-β activation may explain the observed phenotype of enhanced TGF-β activation in asthmatic ASM cells we applied cyclical mechanical stretch. Stretching caused increased p-Smad2 in both asthmatic and nonasthmatic ASM cells (supplementary figure S2b and c). We found no significant difference in p-Smad2 levels in stretched asthmatic ASM cells compared with nonasthmatic ASM cells (supplementary figure S2b and c). We concluded that while contraction and cytoskeletal alterations affect basal TGF-β activation, these factors were unlikely to be solely responsible for driving enhanced TGF-β activation in asthmatic ASM cells.

We hypothesised that ECM alterations could be an additional factor driving increased TGF-β activation in asthmatic ASM cells. We performed ECM crossover experiments using decellularised ECM preparations to investigate whether ECM could drive changes in basal TGF-β activation. Culturing nonasthmatic ASM cells on asthmatic cell-derived ECM increased TGF-β activation compared with culturing on their own endogenous ECM (denoted by the blue dotted line in figure 2e). Similarly, culturing asthmatic ASM cells on nonasthmatic ECM led to a significant decrease in TGF-β activation compared with culturing on their own endogenous ECM (denoted by the blue dotted line in figure 2e). The magnitude of effect of opposite disease state ECM on cells was greater in asthmatic ASM cells *versus* nonasthmatic cells (p=0.0159) (supplementary figure S2c).

### ECM cross-linking enzyme LOXL2 is increased in asthma and regulates ECM stiffness

**FIGURE 3:**
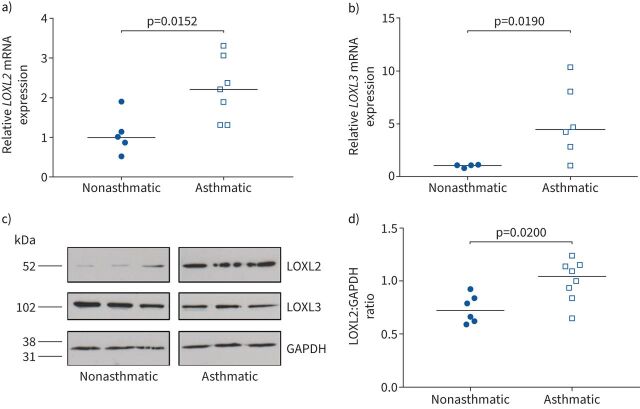
Lysyl oxidase like 2 (LOXL2) expression is increased in asthmatic airway smooth muscle (ASM) cells. a, b) Expression of a) *LOXL2* and b) *LOXL3* mRNA was determined by quantitative reverse transcriptase-PCR. Data are presented as median fold change *versus* mean of the nonasthmatic group. The nonparametric Mann–Whitney test was used. c) Representative Western blots for LOXL2, LOXL3 and GAPDH in nonasthmatic (n=3) and asthmatic (n=3) ASM cells. d) Densitometric analysis of Western blots for LOXL2 in c). Data are presented as median LOXL2:GAPDH ratio. The nonparametric Mann–Whitney test was used.

To investigate a mechanism driving alterations in asthmatic ECM we assessed expression of ECM cross-linking enzymes. *LOXL2* and *LOXL3* mRNAs were significantly increased in asthmatic ASM cells compared with controls (figure 3a and b). There was also a trend towards increased *TGM2*, *LOX* and *LOXL4* expression, but no difference in *LOXL1* expression (supplementary figure S3a–d). We sought to confirm increased *LOXL2* and *LOXL3* at the protein level (figure 3c). LOXL2 protein expression was increased in asthmatic ASM cells compared with nonasthmatic cells (figure 3c and d). Despite increased levels of *LOXL3* mRNA (figure 3b), expression of LOXL3 protein was reduced in asthmatic ASM cells (figure 3c and supplementary figure S3e).

**FIGURE 4:**
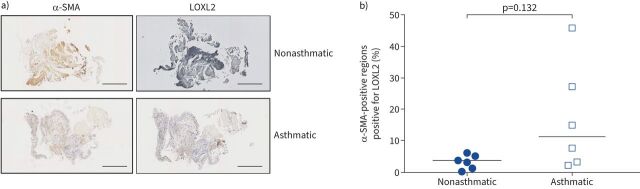
Lysyl oxidase like 2 (LOXL2) is increased in asthmatic bronchial biopsies. a) Representative images demonstrating α-smooth muscle actin (α-SMA) and LOXL2 immunostaining in parallel sections from biopsies from a nonasthmatic and an asthmatic donor. Scale bar: 500 μm. b) Quantification of LOXL2 staining in human bronchial biopsies described in a) shown as percentage of α-SMA-positive regions that were positive for LOXL2. The nonparametric Mann–Whitney test was used.

To further investigate whether LOXL2 expression is increased in asthma we stained parallel sections of airway biopsies from nonasthmatic and asthmatic donors for both α-SMA and LOXL2 (figure 4a). We quantified the percentage of smooth muscle (α-SMA-positive) within the biopsy that was positive for LOXL2 (figure 4b). Our data demonstrated variability in LOXL2 positivity within smooth muscle bundles of asthmatic biopsies, including high levels that were not observed in nonasthmatic controls (figure 4b). LOXL2 positivity was also observed in non-α-SMA-positive cell populations present within the biopsies in both asthmatic and nonasthmatic biopsies (figure 4a).

### LOXL2 inhibition reduces ECM stiffness and allergen-induced airway remodelling

**FIGURE 5:**
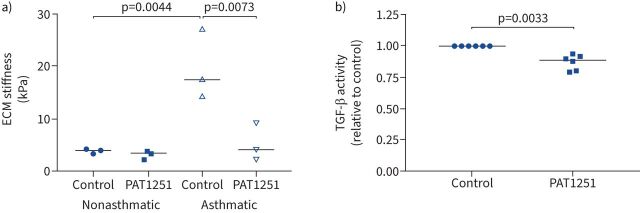
Lysyl oxidase like 2 (LOXL2) inhibition affects airway smooth muscle (ASM) matrix stiffness *in vitro*. a) Atomic force microscopy of extracellular matrix (ECM) deposited cells cultured with LOXL2 inhibitor (PAT1251; 1 µM) or dimethyl sulfoxide (DMSO) vehicle control. Data are presented as median ECM stiffness (kPa). The Kruskal–Wallis nonparametric test with Dunn's multiple comparison test was used. b) Asthmatic ASM cells were cultured for 3 days with LOXL2 inhibitor (PAT1251; 1 µM) or DMSO control and then TGF-β activity assessed. Data are presented as median TGF-β activity relative to DMSO control. The one-sample t-test was used.

To investigate the effect of LOXL2 on the stiffness of ECM deposited by ASM cells we treated ASM cells with a LOXL2 inhibitor (PAT1251; median inhibitory concentration (IC_50_) 0.71 µM). Treatment with 1 µM PAT1251 reduced the stiffness of ECM deposited by asthmatic ASM cells to an equivalent stiffness as that deposited by nonasthmatic controls (figure 5a). Furthermore, 1 µM PAT1251 led to a small but significant reduction in basal TGF-β activation by asthmatic cells (figure 5b).

**FIGURE 6:**
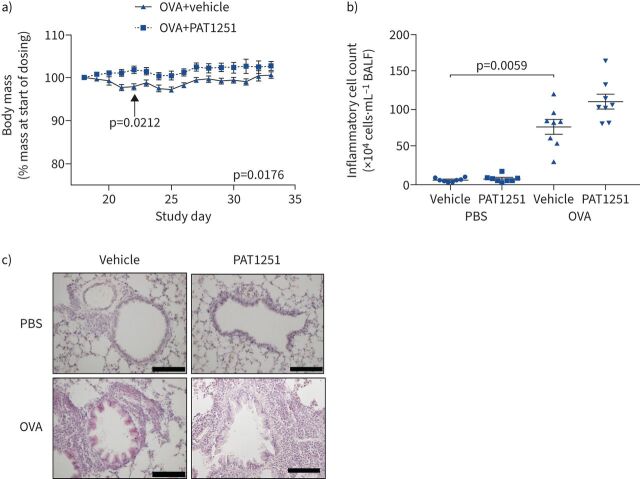
Lysyl oxidase like 2 (LOXL2) inhibition *in vivo* protects mice against ovalbumin (OVA) challenge-induced weight loss. a) Body mass data from mice challenged with OVA and treated with either vehicle control or LOXL2 inhibitor (PAT1251; 30 mg·kg^−1^). Data are presented as median with interquartile range. Two-way ANOVA was used. b) The total number of bronchoalveolar lavage fluid (BALF) inflammatory cells was quantified and presented as mean±sem number of inflammatory cells. The Kruskal–Wallis nonparametric test with Dunn's multiple comparison test was used. c) Representative images of haematoxylin/eosin-stained lung tissue. Images are representative of n=8 animals per group. Scale bar: 100 μm.

We next investigated a role for LOXL2 in OVA-induced airway remodelling *in vivo*. Daily oral dosing with 30 mg·kg^−1^ PAT1251 [18] from the start of OVA challenges protected mice from OVA challenge-related decreases in body mass (figure 6a). There was no significant difference in starting body mass between treatment groups (supplementary figure S4a). OVA challenge led to increased bronchoalveolar lavage inflammation (figure 6b and c), including increased percentages of eosinophils, lymphocytes and neutrophils, and a concomitant decrease in macrophages (supplementary figure S4b–e). PAT1251 had no significant effect on OVA challenge-induced airway inflammation (figure 6b and c) nor did it affect the differential cell counts of eosinophils, lymphocytes, neutrophils or macrophages (supplementary figure S4b–e).

**FIGURE 7:**
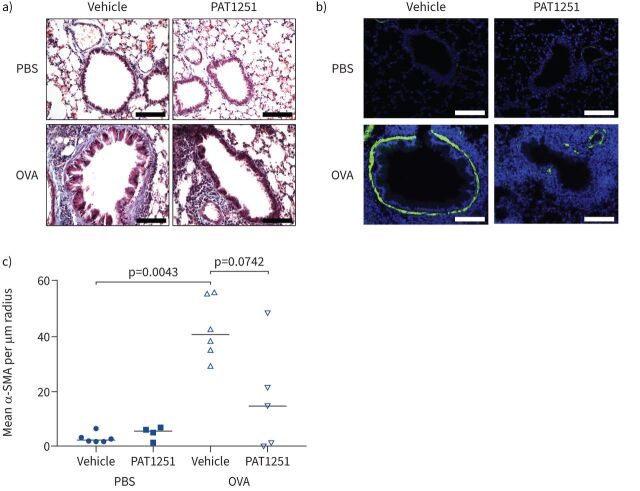
Lysyl oxidase like 2 inhibition *in vivo* reduces chronic ovalbumin (OVA) challenge-induced airway remodelling. a) Representative images of Masson's trichrome-stained lung tissue. Images are representative of n=8 animals per group. Scale bar: 100 μm. b) Representative images of α-smooth muscle actin (α-SMA) and 4′,6-diamidino-2-phenylindole-stained lung tissue. Images are representative of the following group sizes: PBS+vehicle control n=8, PBS+PAT1251 n=7, OVA+vehicle n=7 and OVA+PAT1251 n=8. Scale bar: 100 μm. A negative control image is shown in supplementary figure S4. c) α-SMA staining around small airways (<100 µm radius) was quantified. Data are presented as mean α-SMA area per µm radius. The Kruskal–Wallis nonparametric test with Dunn's multiple comparison test was used.

OVA challenge caused increased subepithelial collagen deposition compared with saline control-challenged animals as shown by Masson's trichrome staining (figure 7a). The amount of collagen surrounding airways in PAT1251-treated OVA-challenged animals was reduced compared with OVA-challenged vehicle controls (figure 7a). This was supported by polarised light microscopy of picrosirius red staining lung tissue, which showed that treatment with PAT1251 reduced total amounts of collagen around airways and the organisation of collagen fibres, as shown by a shift in colours observed from a combination of red and yellow in vehicle control-treated animals to solely red in PAT1251-treated animals (supplementary figure S4g). Furthermore, OVA challenge caused thickening of the ASM layer around small airways (<100 µm radius) (figure 7b and c). We demonstrated a >2-fold decrease in the ASM thickness in PAT1251-treated animals compared with controls (figure 7c).

## Discussion

The process of fibrogenesis and tissue remodelling is driven by ECM stiffness in multiple tissues and organs [7]. In severe asthma, airway remodelling is associated with subepithelial fibrosis and thickening of the ASM layer, both of which may increase airway wall stiffness [12]. We have investigated how ECM influences TGF-β activation in ASM cells, and identified a novel and important role for the matrix cross-linking enzyme LOXL2 in asthmatic airway remodelling.

TGF-β is a key driver of asthmatic airway remodelling and we have previously shown that ASM cells activate TGF-β in response to contractile agonists [21]. Here, we demonstrate for the first time that basal TGF-β activation by ASM cells is affected by both ECM stiffness and by cyclical stretch, confirming earlier studies in other cell types [30, 32]. Due to the confirmatory nature of these studies we used a relatively small number of three ASM donor cell lines in order to preserve valuable primary human ASM cell cultures for hypothesis-driven exploratory experiments in the rest of the article. Given our previous findings and that of multiple other research groups [21, 30, 33], we would hypothesise that the influence of ECM stiffness and cyclical stretch on ASM cell TGF-β activation is mediated by cell surface integrins; however, a role for other mechanisms including proteases [34] cannot be discounted based upon the present studies.

We found that asthmatic ASM cells are more contractile than nonasthmatic cells when measured using a collagen gel contraction assay and TGF-β activation correlates with contractility, confirming previously studies [35]. However, we found no difference in contractility between asthmatic and nonasthmatic cells when TFM was utilised. This is potentially explained by the inherent differences in the two assays. Collagen gels result in a three-dimensional culture of ASM cells, whereas TFM is performed on a two-dimensional monolayer. Crucially for our data interpretation, the weak correlation between TGF-β activation and collagen gel contractility combined with the TFM data, which more closely resembles the two-dimensional nature of TMLC co-culture assays, led us to conclude that cell contractility differences are not responsible for increased TGF-β activation in asthmatic ASM cells.

It has long been postulated that the airway wall is stiffer in asthma; however, very few studies have directly investigated matrix stiffness in asthma. The passive stiffness of asthmatic smooth muscle explants is increased following length perturbations [36] and asthmatic fibroblasts have a higher Young's elastic modulus than nonasthmatic controls [37]. To the best of our knowledge, this study is the first to directly measure the stiffness of ASM cell-derived ECM and show that asthmatic ASM cells deposit stiffer ECM.

The ECM influences a variety of cellular pathways, and can affect ASM cell biology [4, 5, 38–40] and impact bronchoconstriction [41, 42]. The data presented here demonstrate a link between increased ASM cell ECM stiffness and asthmatic airway remodelling *via* TGF-β activation. While there is a paucity of data mechanistically linking increased ECM stiffness with airway remodelling, matrix stiffening activates pulmonary artery smooth muscle to trigger blood vessel remodelling [43, 44].

ECM stiffness is driven in part through cross-linking of matrix proteins. It has previously been reported that ASM cells can remodel extracellular collagen fibres [45]. LOXL2 is a cross-linking enzyme that has been investigated as a druggable target in fibrotic diseases [46]. Our data confirm an earlier study showing increased *LOXL2* gene expression in asthma [47], but also demonstrate that LOXL2 protein is increased in asthmatic ASM. LOXL2 is increased in several diseases associated with tissue remodelling [13–17, 46], but the functional consequences of increased LOXL2 in asthma have never been studied. Our data suggest heterogeneity in LOXL2 expression between donors, which may be reflective of disease severity; however, the current study was not sufficiently powered to detect associations between LOXL2 expression and asthma severity. The mechanisms driving enhanced LOXL2 expression in asthma are likely to be complex and multifactorial, and may potentially include transcriptional regulation, microRNAs, alternative splicing and/or epigenetic regulation. Developing a greater understanding of these underlying mechanisms is clearly an important area for future research; however, it is beyond the scope of this article.

We acknowledge that changes in LOXL2 expression do not necessarily lead to changes in enzymatic activity; however, there is currently no commercially available assay to measure LOXL2 activity. We therefore sought to investigate the functional effects of inhibiting LOXL2 activity *in vitro* and *in vivo*. Our findings suggest that LOXL2 increases the stiffness of ASM cell ECM, driving TGF-β activation and remodelling. Specifically, LOXL2 inhibition *in vivo* reduced allergen-induced airway remodelling as measured by collagen deposition around airways and ASM layer thickening. This is the first report linking LOXL2 with airway remodelling, but previous studies link LOXL2 to vascular remodelling *via* effects on smooth muscle [48]. It is important to note that while animal models of fibrosis have shown promise for LOXL2 inhibition [17–20, 49], this has not yet translated to a clinical benefit in patients [50]. The reasons for this are unclear and there is merit in further consideration of LOXL2 inhibition in the treatment of asthmatic airway remodelling.

We have focused upon quantifying ASM layer thickness in smaller airways <100 µm in radius in line with our previous work [21]. While airway remodelling changes are likely to affect airways of all sizes, we and others have found that the largest remodelling changes occur in small airways compared with large airways [21, 51, 52]. Furthermore, hyperpolarised gas magnetic resonance imaging of the lung has demonstrated significant regional heterogeneity in gas ventilation and distribution, suggestive of differential effects in smaller airways [53].

In addition to measuring ASM layer thickness we used two histological stains to demonstrate that LOXL2 inhibition *in vivo* reduces deposition of collagens around airways. The use of polarised light microscopy to visualise picrosirius red-stained lung tissue demonstrated reduced collagen around the airways of OVA-challenged animals treated with a LOXL2 inhibitor compared with vehicle control-treated animals. Crucially, the observed colour of collagen fibres was altered between the two groups of animals, with a mixture of reds, oranges and yellows present in vehicle control-treated animals compared with predominantly red fibres in animals treated with a LOXL2 inhibitor. This is suggestive of a change in collagen fibre organisation and orientation [54], consistent with altered collagen maturation.

In addition to altering matrix cross-linking, LOXL2 has recently been shown to have both transcriptional and epigenetic regulatory functions [55]. Changes in transcriptional and epigenetic regulation have long been associated with asthma pathogenesis [56, 57]. This raises the possibility that increased LOXL2 expression in asthma might impact a large number of pathways relevant to asthma pathogenesis that are independent of its matrix cross-linking enzymatic activity. Such pathways might include immune and inflammatory pathways in addition to the data shown here demonstrating a role for LOXL2 in asthmatic airway remodelling events. This warrants further investigation in future studies.

The data presented here highlight a potentially important role for LOXL2 in the development of asthmatic airway remodelling. We show that increased ECM can drive TGF-β activation in ASM cells and demonstrate that LOXL2 contributes to ECM stiffness. We demonstrate for the first time that LOXL2 expression is increased in asthma and we have shown that LOXL2 inhibition *in vivo* may reduce airway remodelling. Taken together, our findings suggest that inhibition of LOXL2 may have merit as an approach to reduce airway remodelling in severe asthma.

## Supplementary material

10.1183/13993003.04361-2020.Supp1 **Please note:** supplementary material is not edited by the Editorial Office, and is uploaded as it has been supplied by the author.
Supplementary methods ERJ-04361-2020.Methods
Supplementary figure S1. a) GelMa substrates (5, 10 and 15% GelMa wt/vol) were subjected to compressive modulus using texture analyser to determine the relative stiffness. 5% GelMa was approximately equivalent to the described stiffness of tracheal smooth muscle [22] and so was denoted as 1x stiffness. b) *TGFB1* mRNA was determined by qRT-PCR. Data is expressed as median fold change *versus* mean of the non-asthmatic group. Non-parametric Mann Whitney test was used. c) *TGFB2* mRNA was determined by qRT-PCR. Data is expressed as median fold change *versus* mean of the non-asthmatic group. Non-parametric Mann Whitney test was used. d) *TGFB3* mRNA was determined by qRT-PCR. Data is expressed as median fold change *versus* mean of the non-asthmatic group. Non-parametric Mann Whitney test was used. ERJ-04361-2020.Figure_S1
Supplementary figure S2. a) Basal traction force was determined using traction force microscopy. Data is presented as median root mean square traction (n) across all donor cell lines tested. Non-parametric Mann Whitney test was used. b) ASM cells were subjected to stretch (15%, 0.3Hz (S)) or left unstretched (U) for 4 h. PSmad2, total Smad2/3 and GAPDH were measured by western blotting. Figure shown is representative of n=6 non-asthmatic and n=6 asthmatic donor cell lines. Densitometrical analysis of all blots was performed and data is expressed as ratio PSmad2:tSmad2/3. Kruskall Wallis non-parametric test with Dunn’s multiple comparison test was used. c) Data from figure 2e are expressed here in a format that allows comparison of the effect of ECM on basal TGF-β activity for both non-asthmatic and asthmatic ASM cells. Mann Whitney was used to test for a difference between the ECM effect delta between non-asthmatic and asthmatic cells (p=0.0159). ERJ-04361-2020.Figure_S2
Supplementary figure S3. a) *TGM2* mRNA in ASM cells was determined by qRT-PCR. Data is expressed as median fold change *versus* the mean data from the non-asthmatic group. Non-parametric Mann Whitney test was used, b) *LOX* mRNA in ASM cells was determined by qRT-PCR. Data is expressed as median fold change *versus* the mean data from the non-asthmatic group. Non-parametric Mann Whitney test was used. c) *LOXL1* mRNA in ASM cells was determined by qRT-PCR. Data is expressed as median fold change *versus* the mean data from the non-asthmatic group. Non-parametric Mann Whitney test was used. d) *LOXL4* mRNA in ASM cells was determined by qRT-PCR. Data is expressed as median fold change *versus* the mean data from the non-asthmatic group. Non-parametric Mann Whitney test was used. e) Densitometrical analysis of LOXL3 western blots (figure 3c). Data is expressed as median ratio LOXL3:GAPDH. Non-parametric Mann Whitney test was used. ERJ-04361-2020.Figure_S3
Supplementary figure S4. a) Starting body mass data from mice in the *in vivo* model of asthma. Non-parametric Kruskall-Wallis with Dunn’s multiple comparison test was used. b) The median percentage of eosinophils within the inflammatory cell population of BALF (figure 6b) was determined by differential cell count. Non-parametric Kruskall-Wallis with Dunn’s multiple comparison test was used. c) The median percentage of neutrophils within the inflammatory cell population of BALF (figure 6b) was determined by differential cell count. Non-parametric Kruskall-Wallis with Dunn’s multiple comparison test was used. d) The median percentage of lymphocytes within the inflammatory cell population of BALF (figure 6b) was determined by differential cell count. Non-parametric Kruskall-Wallis with Dunn’s multiple comparison test was used. e) The median percentage of macrophages within the inflammatory cell population of BALF (figure 6b) was determined by differential cell count. Non-parametric Kruskall-Wallis with Dunn’s multiple comparison test was used. f) Representative negative control image for α-SMA immunofluorescence staining in figure 7b. g) Representative images of picro-Sirius red stained mouse lung tissue from the ovalbumin model. Tissue from animals treated with vehicle control was imaged using polarised light microscopy (i) and under brightfield light (ii). Similarly, tissue from animals treated with the LOXL2 inhibitor (PAT1251) was imaged using polarised light microscopy (i) and under brightfield light (ii). ERJ-04361-2020.Figure_S4
Supplementary table S1. Table detailing materials used in the manuscript, including suppliers and clone details of all antibodies used. ERJ-04361-2020.Table_S1
Supplementary table S2. Table describing primer sequences for qRT-PCR. All primers were designed using Primer3 online software. ERJ-04361-2020.Table_S2

## Shareable PDF

10.1183/13993003.04361-2020.Shareable1 This one-page PDF can be shared freely online.
Shareable PDF ERJ-04361-2020.Shareable
