# Supplementary material for: Lysyl oxidase like 2 is increased in asthma and contributes to asthmatic airway remodelling
Source: Eur Respir J. 2022 Jul 7;60(1):2004361. doi: 10.1183/13993003.04361-2020 (PMC9260127; doi:10.1183/13993003.04361-2020)
Supplement: Supplementary file 1 [file ERJ-04361-2020.Methods.pdf]

Supplemental Methods

Cell culture

Human ASM cells were cultured from bronchial biopsies obtained at the Nottingham Respiratory Biomedical Research Unit, University of Nottingham (ethics number 08/H0407/1) and the Institute for Lung Health, University of Leicester (ethics number 08/H0406/189). Biopsies were obtained with written, informed consent from non-asthmatic and asthmatic donors. ASM cells were cultured from biopsy explants and used at passage 6. The cells were cultured in Dulbecco's modified Eagles medium (DMEM) supplemented with 10% foetal bovine serum and 4mM L-glutamine at 37°C, 5% CO_2 in a humidified incubator. Cells were growth arrested in serum-free DMEM for 24 hours prior to all experiments. A minimum of 3 donor cell lines were used in each experiment; in all experiments n is shown in the figures.

Human Bronchial Biopsy Collection

Bronchial biopsies obtained from stable asthmatic and healthy control donors (n=6 per group) were used to determine the expression of LOXL2 protein within smooth muscle bundles. All tissue was collected at the Nottingham Biomedical Research Centre, University of Nottingham, under informed, written consent with ethical approval (12/EM/0199). Biopsies were obtained from 2nd subdivision of right main bronchus. The asthmatic donors all had a formal diagnosis of asthma made by a medical practitioner, no history of exacerbation for 6 weeks prior to the bronchoscopy, and no recent use of oral corticosteroids, phosphodiesterase inhibitors or leukotriene inhibitors. Bronchial biopsies were fixed in formalin for 24 hours then paraffin wax embedded. The biopsies used have been previously published in [3]

LOXL2 Inhibition in an In Vivo Ovalbumin Model

Studies were approved by the University of Nottingham Animal and Welfare Ethical Review Board (AWERB) and performed under Home Office personal, project and institutional license authority within the Animal (Scientific Procedures) Act 1986. Animals received free access to food (Tekland Global 18% protein rodent diet) and water, and were housed in specific pathogen free environment. 6-week-old female Balb/C mice (Charles River, UK) were sensitised to ovalbumin by intraperitoneal injection (i.p) of 10μg ovalbumin (Sigma Aldrich, UK) diluted 1:1 with Alum on days 0 and 12. Following sensitisation to ovalbumin animals were randomised to one of four treatment groups PBS + vehicle, PBS + PAT1251, OVA + vehicle, OVA + PAT1251. From day 18 animals received daily oral gavage of either 30mg/kg LOXL2 inhibitor (PAT1251; Med Chem Express, China) in 0.5% methylcellulose or 0.5% methylcellulose alone (100μl/dose). Animals were challenged with either 400

μg/ml ovalbumin in 50μl PBS or 50μl PBS alone via the oropharyngeal route under isoflurane anaesthesia on days 19, 20, 21, 22, 23, 24, 26, 28, 30 and 33. Animals were euthanised 24 hours after the final challenge. Bronchoalveolar lavage (BAL) wash performed using 1ml PBS. BAL inflammatory cells were cytospun (400rpm for 6 minutes) and stained with RapiDiff II kit (Atom Scientific, UK) according to the manufacturer's instructions. A differential cell count of eosinophils, monocytes, neutrophils and leukocytes was performed using a Nikon Eclipse 90i microscope. Total BAL inflammatory cell count was performed using a haemocytometer.

Statistical Analysis

All statistical tests were discussed with and approved by a statistician (I.D.S). Data are reported as a median of n observations and non-parametric tests were chosen due to the relatively small sample sizes restricting assumptions of a normal distribution and so that tests were not affected by outliers. Mann-Whitney or Kruskall-Wallis tests were used to compare two, or more than two groups, respectively. All ECM crossover experiments were analysed using a one-sample t-test versus the effect on the cells' own ECM. Details of the specific statistical test used for each figure is included in the figure legend. In all in vivo studies the experimental unit (n) denotes an individual animal. All analysis was performed using Graphpad Prism (v7.04, La Jolla, USA)
