# Supplementary figures and images for: Lysyl oxidase like 2 is increased in asthma and contributes to asthmatic airway remodelling
Source: Eur Respir J. 2022 Jul 7;60(1):2004361. doi: 10.1183/13993003.04361-2020 (PMC9260127; doi:10.1183/13993003.04361-2020)

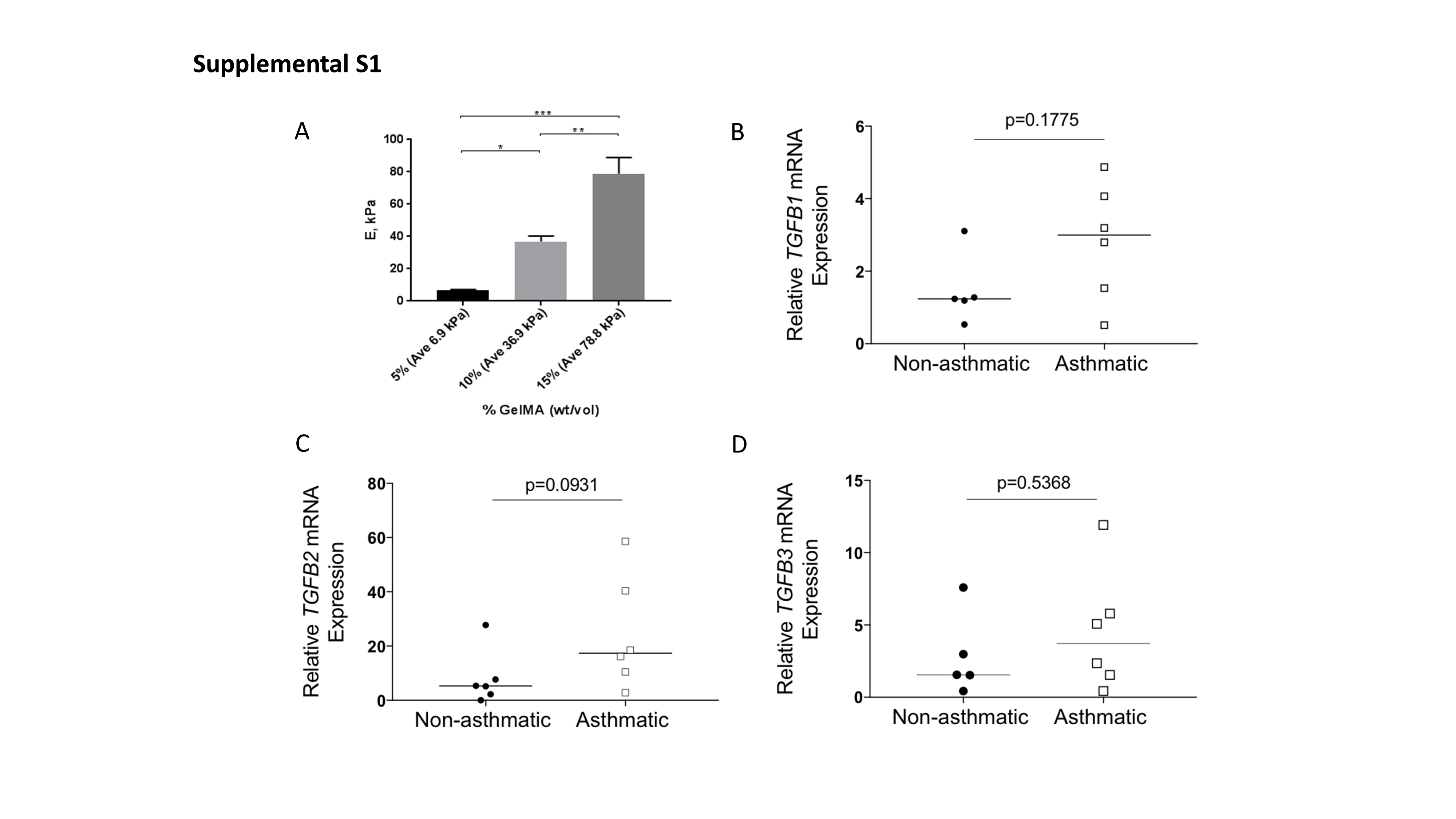

Supplement: Supplementary file 2 [file ERJ-04361-2020.Figure_S1.tif]

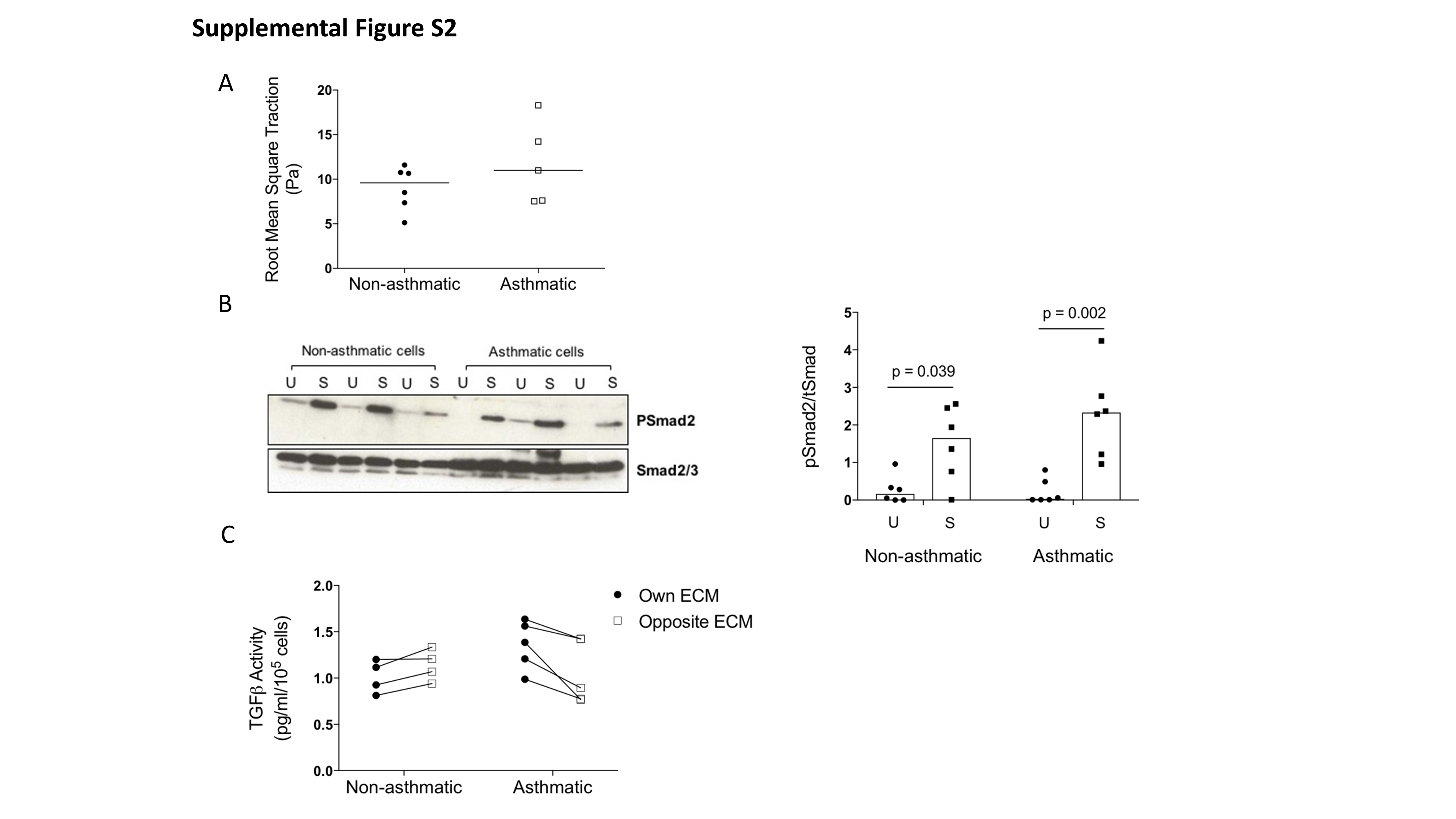

Supplement: Supplementary file 3 [file ERJ-04361-2020.Figure_S2.tif]

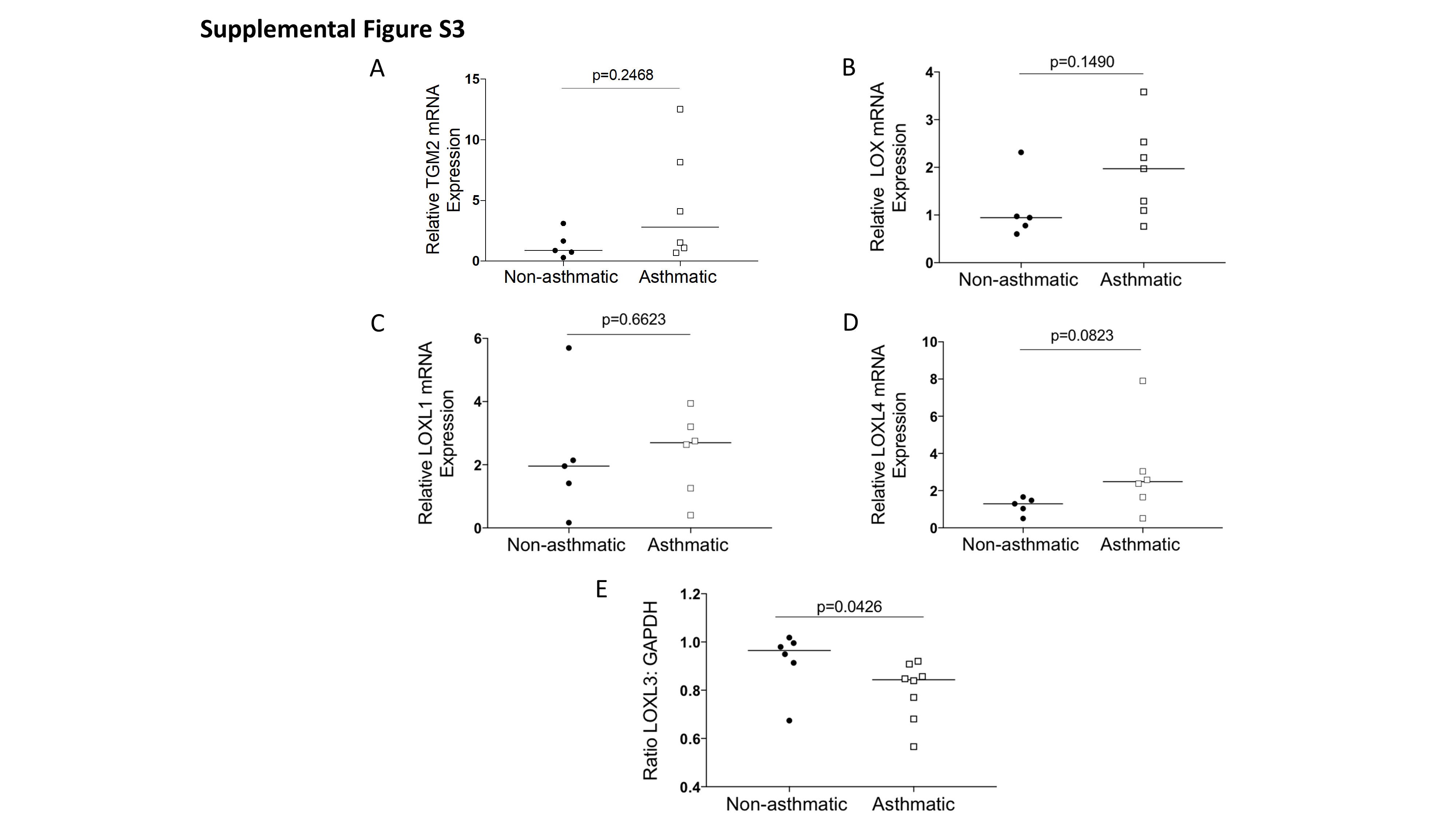

Supplement: Supplementary file 4 [file ERJ-04361-2020.Figure_S3.tif]

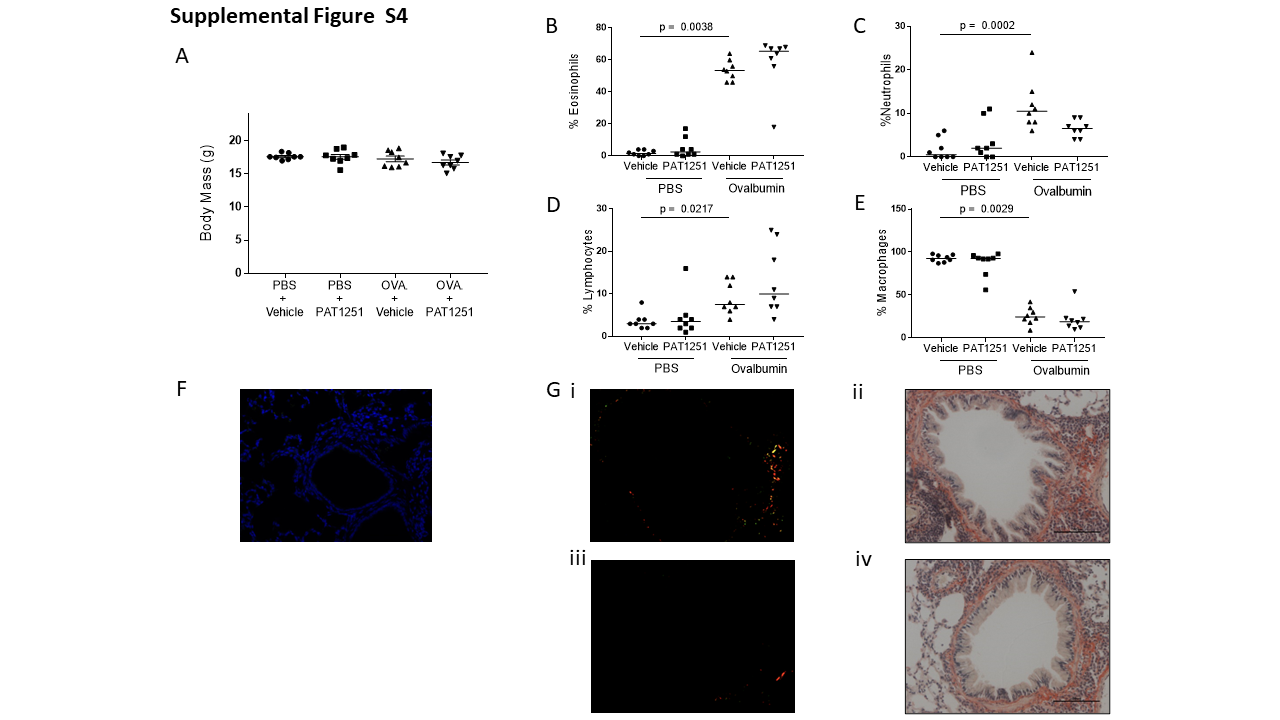

Supplement: Supplementary file 5 [file ERJ-04361-2020.Figure_S4.tif]

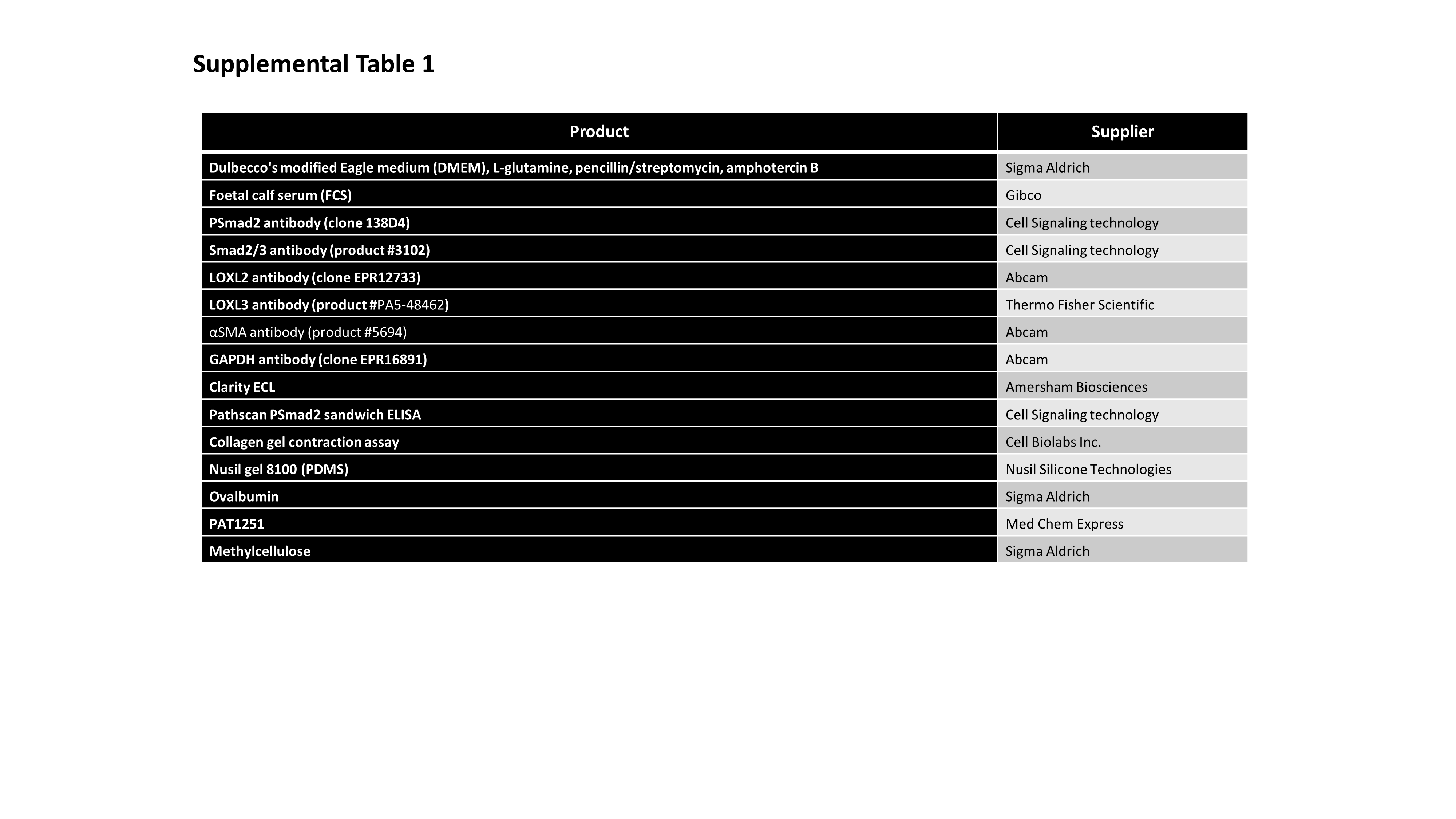

Supplement: Supplementary file 6 [file ERJ-04361-2020.Table_S1.tif]

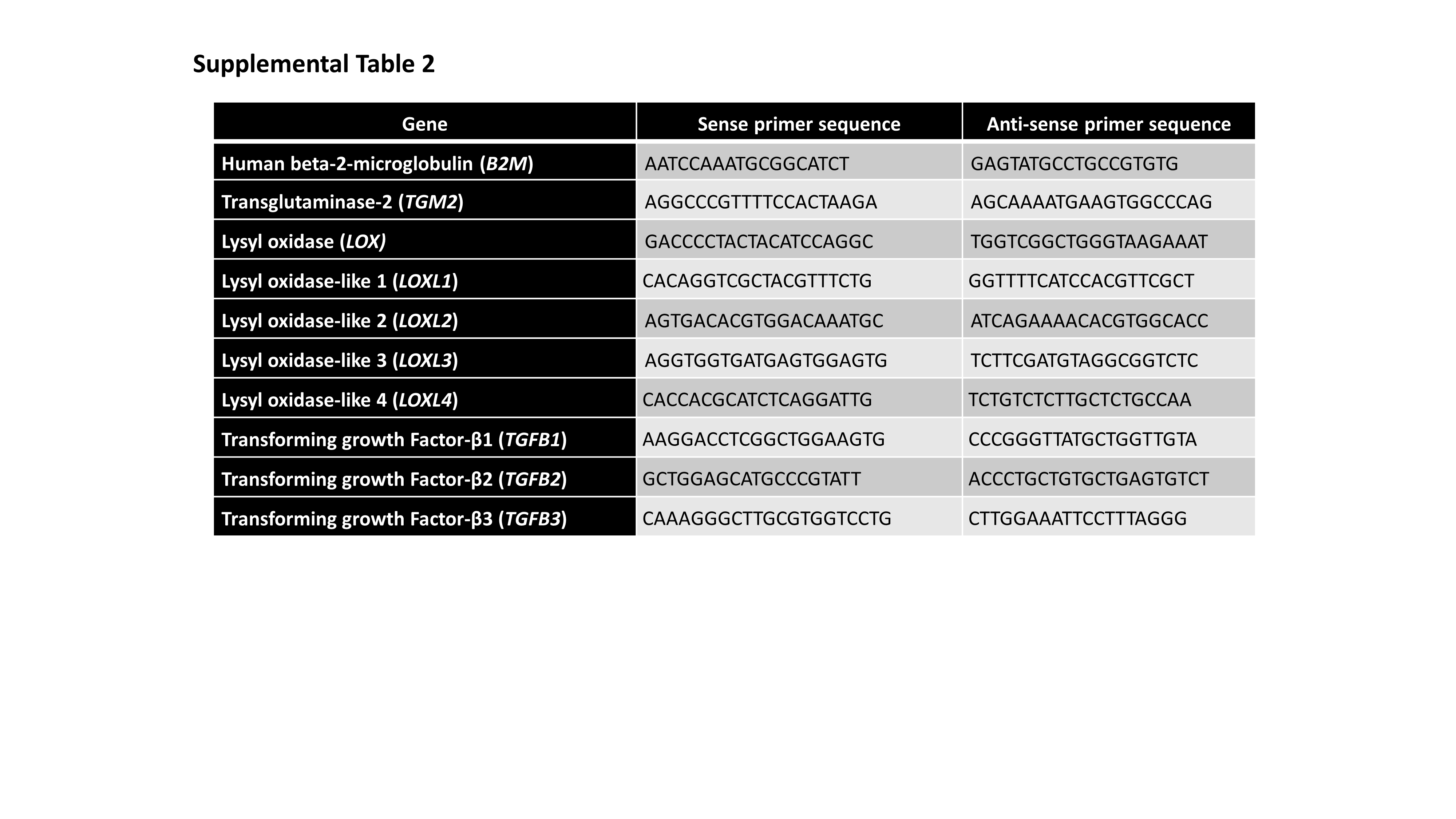

Supplement: Supplementary file 7 [file ERJ-04361-2020.Table_S2.tif]
