# Supplementary material for: Lysyl oxidase like 2 is increased in asthma and contributes to asthmatic airway remodelling
Source: Eur Respir J. 2022 Jul 7;60(1):2004361. doi: 10.1183/13993003.04361-2020 (PMC9260127; doi:10.1183/13993003.04361-2020)
Supplement: Supplementary file 8 [file ERJ-04361-2020.Shareable.pdf]

EUROPEAN RESPIRATORY JOURNAL
ORIGINAL RESEARCH ARTICLE
J. RAMIS ET AL.

SHAREABLE PDF

Lysyl oxidase like 2 is increased in asthma and contributes to asthmatic airway remodelling

Jopeth Ramis[1,2], Robert Middlewick[3], Francesco Pappalardo[1], Jennifer T. Cairns[3], Iain D. Stewart[3,4], Alison E. John[3,4], Shams-Un-Nisa Naveed[3,5], Ramaswamy Krishnan[6], Suzanne Miller[1,3], Dominick E. Shaw[3], Christopher E. Brightling[5], Lee Buttery[1], Felicity Rose[1], Gisli Jenkins[3,4], Simon R. Johnson[1,3] and Amanda L. Tatler[3]

[1]Biodiscovery Institute, University of Nottingham, Nottingham, UK. [2]Dept of Chemical Engineering, Technological Institute of the Philippines, Manila, Philippines. [3]Centre for Respiratory Research/NIHR Biomedical Research Centre, School of Medicine, University of Nottingham, Nottingham, UK. [4]Margaret Turner Warwick Centre for Fibrosing Lung Disease, National Heart and Lung Institute, Imperial College London, London, UK. [5]Institute for Lung Health, Leicester NIHR Biomedical Research Centre, University of Leicester, Leicester, UK. [6]Center for Vascular Biology Research, Beth Israel Deaconess Medical Center, Harvard Medical School, Boston, MA, USA.

Corresponding author: Amanda L. Tatler (amanda.tatler@nottingham.ac.uk)

Check for updates

Shareable abstract (@ERSpublications)
Novel role for matrix cross-linking enzyme LOXL2 in asthmatic airway remodelling: LOXL2 is increased in #asthma but LOXL2 inhibition reduces matrix stiffness in airway smooth muscle cells and reduces remodelling *in vivo* https://bit.ly/3FnzGb3

Cite this article as: Ramis J, Middlewick R, Pappalardo F, *et al*. Lysyl oxidase like 2 is increased in asthma and contributes to asthmatic airway remodelling. *Eur Respir J* 2022; 60: 2004361 [DOI: 10.1183/13993003.04361-2020].

This single-page version can be shared freely online.

Copyright ©The authors 2022.

This version is distributed under the terms of the Creative Commons Attribution Licence 4.0.

Received: 30 Nov 2020
Accepted: 8 Nov 2021

Abstract

Background Airway smooth muscle (ASM) cells are fundamental to asthma pathogenesis, influencing bronchoconstriction, airway hyperresponsiveness and airway remodelling. The extracellular matrix (ECM) can influence tissue remodelling pathways; however, to date no study has investigated the effect of ASM ECM stiffness and cross-linking on the development of asthmatic airway remodelling. We hypothesised that transforming growth factor-β (TGF-β) activation by ASM cells is influenced by ECM in asthma and sought to investigate the mechanisms involved.

Methods This study combines *in vitro* and *in vivo* approaches: human ASM cells were used *in vitro* to investigate basal TGF-β activation and expression of ECM cross-linking enzymes. Human bronchial biopsies from asthmatic and nonasthmatic donors were used to confirm lysyl oxidase like 2 (LOXL2) expression in ASM. A chronic ovalbumin (OVA) model of asthma was used to study the effect of LOXL2 inhibition on airway remodelling.

Results We found that asthmatic ASM cells activated more TGF-β basally than nonasthmatic controls and that diseased cell-derived ECM influences levels of TGF-β activated. Our data demonstrate that the ECM cross-linking enzyme LOXL2 is increased in asthmatic ASM cells and in bronchial biopsies. Crucially, we show that LOXL2 inhibition reduces ECM stiffness and TGF-β activation *in vitro*, and can reduce subepithelial collagen deposition and ASM thickness, two features of airway remodelling, in an OVA mouse model of asthma.

Conclusion These data are the first to highlight a role for LOXL2 in the development of asthmatic airway remodelling and suggest that LOXL2 inhibition warrants further investigation as a potential therapy to reduce remodelling of the airways in severe asthma.
